# Ursolic-Acid-Enriched Herba Cynomorii Extract Protects against Oxidant Injury in H9c2 Cells and Rat Myocardium by Increasing Mitochondrial ATP Generation Capacity and Enhancing Cellular Glutathione Redox Cycling, Possibly through Mitochondrial Uncoupling

**DOI:** 10.1155/2013/924128

**Published:** 2013-04-10

**Authors:** Jihang Chen, Kam Ming Ko

**Affiliations:** Division of Life Science, Hong Kong University of Science & Technology, Clear Water Bay, Hong Kong

## Abstract

Mitochondrial decay is considered to be a major contributor to aging-related diseases, including neurodegenerative diseases, cardiovascular disorders, and certain metabolic diseases. Therefore, the maintenance of mitochondrial functional capacity and antioxidant status should play an essential role in preventive health. Herba Cynomorii, which is one of the most potent “Yang-invigorating” Chinese tonic herbs, was found to increase mitochondrial ATP generation capacity (ATP-GC) in rat hearts *ex vivo*. In the present study, we demonstrated that HCY2, an active fraction of Herba Cynomorii, and its major ingredient ursolic acid (UA) could protect against hypoxia/reoxygenation-induced cell apoptosis in H9c2 cells *in vitro* and also against ischemia/reperfusion-induced injury in rat hearts *ex vivo*. The cardioprotection was associated with an increase in ATP-GC and an enhancement of glutathione redox cycling. The results suggest that UA may be one of the active ingredients responsible for the cardioprotection afforded by Herba Cynomorii, and this effect may be mediated, at least in part, by enhancement of mitochondrial functional capacity and antioxidant status, possibly through the induction of mitochondrial uncoupling.

## 1. Introduction

Aging is defined as an inevitable degenerative process in physiological functions and metabolic processes, chronologically leading to morbidity and mortality [1]. Emerging evidence has linked mitochondrial decay to a variety of age-related diseases, including, but not limited to, neurodegenerative diseases, cardiovascular disorders, and cancer [2]. The investigation of mitochondrial function in various organs of aging animals has shown a variety of mitochondrial alterations [3, 4] that point to defects at specific sites in mitochondrial energy transfer pathways, for example, the respiratory chain [5, 6], which result in age-dependent decreases in mitochondrial ATP generation capacity (ATP-GC) [7, 8]. This age-associated deterioration of mitochondrial bioenergetics is invariably accompanied by progressive impairment in mitochondrial antioxidant status [7, 9]. Given the central role of mitochondria in the aging process, the maintenance of mitochondrial functional capacity and antioxidant status is important for preventive health, especially for organs having a major requirement for energy in the form of ATP, such as the heart [10, 11]. 

Efforts to retard the adverse consequences of aging have long been a preoccupation of mankind, and traditional Chinese medicine (TCM) always emphasizes the prolongation of a healthy lifespan. In this regard, many Chinese tonic herbs have long been used for safeguarding health and delaying the onset of senility. According to TCM theory, tonic herbs, which are used for the treatment of various patterns (Yin, Yang, Qi, Blood) of deficiency in body function, are classified into four categories: Yang-invigorating, Yin-nourishing, Qi-invigorating, and blood-enriching [12]. The blood-enriching and Qi-invigorating herbs are further subgrouped under the Yin family and Yang family, respectively. Holistically, the Yang-invigorating action involves the upregulation of cellular activities, particularly in the heart, which plays a pivotal role in fueling the vital activities in all organs. Previous studies in our laboratory have demonstrated the ability of Yang tonic herbs to increase mitochondrial ATP-GC, which is associated with an increased activity of the mitochondrial electron transport system [13]. In addition to upregulating mitochondrial functional capacity, Yang tonic herbs/formulas have also been shown to enhance cellular/mitochondrial antioxidant status [14] and also decrease mitochondrial coupling efficiency [15], thereby protecting against oxidant injury in various tissues of rats [16, 17].

Herba Cynomorii (the whole plant of *Cynomorium  songaricum* Rupr., Cynomoriaceae), also known as Suo-Yang in Chinese, has been used as a herbal tonic to supplement the primordial “Yang essence” for both men and women in Chinese medicine for hundreds of years. As documented in the Chinese medicine literature, Herba Cynomorii is renowned as “Bu Lao (ageless) herb”, and this bespeaks much of its ability to sustain a youthful life. Preliminary studies in our laboratory have demonstrated that the methanol extract of Herba Cynomorii stimulates mitochondrial ATP-GC in both H9c2 cells *in vitro* and rat hearts *ex vivo*, presumably by increasing mitochondrial electron transport [13, 18]. However, the chemical components responsible for increasing mitochondrial ATP-GC remain to be identified. In addition, it is as yet unclear whether Herba Cynomorii can enhance myocardial mitochondrial antioxidant status and protect the heart against oxidant injury.

In the present study, we endeavored to examine the chemical basis as well as the biochemical mechanism underlying the “Yang-invigorating” action of Herba Cynomorii. The objectives of this study were (1) to isolate the active fraction(s) through bioactivity-guided fractionation, in which the measurement of ATP-GC in H9c2 cells was utilized as an activity monitor; (2) to identify the active principle(s) from active fraction(s); (3) to investigate whether active fraction(s) and their respective active component(s) can afford protection against hypoxia/reoxygenation-induced cell apoptosis in H9c2 cells as well as myocardial ischemia/reperfusion (I/R) injury in rats; and (4) to investigate the possible biochemical mechanism underlying the cardioprotection.

## 2. Materials and Methods

### 2.1. Drugs and Chemicals

Luciferase was obtained from Fluka (Switzerland). Ursolic acid (UA) was purchased from Wako Pure Chemical Industries, Ltd (Japan). Other chemicals were purchased from Sigma Chemical (St Louis, MO). Solvents used for HPLC were of HPLC grade.

### 2.2. Cell Culture

H9c2 cells, a permanent cell line derived from embryonic BD1X rat heart tissue, were purchased from American Type Culture Collection. The cells were cultured as monolayers in Dulbecco's modified Eagle's medium (DMEM) (GIBCO BRL) supplemented with 10% (v/v) fetal bovine serum. The medium contained glucose (4.5 g/L) and glutamine (4.5 mM), supplemented with NaHCO_3_ (17 mM), penicillin (100 IU/mL), and streptomycin (100 *μ*g/mL). All cells were grown under an atmosphere of 5% CO_2_ in air (v/v) at 37°C. The medium was replaced every 2-3 days. A stock of cells was grown in a 75 cm^2^ culture flask and split before confluence at a subcultivation ratio of 1 : 10.

### 2.3. Herbal Material and Extraction

Herba Cynomorii was purchased from a local (Hong Kong-based) herbal dealer (Lee Hoong Kee, Ltd.). The herb was authenticated by the supplier and a voucher specimen (HKUSTY01001) was deposited in the Division of Life Science, the Hong Kong University of Science and Technology (HKUST). For extraction, powdered stems of Herba Cynomorii (14 kg) were extracted with 95% ethanol at an herb-to-solvent ratio of 1 : 6 (w/v) for 2 h under reflux at 70°C. The extraction was repeated once, and the pooled ethanol extracts were filtered and concentrated under reduced pressure using a rotavaporator. The ethanol extraction resulted in a yield of 13% (w/w) with respect to the initial amount of crude herb. The extract was stored at 4°C prior to use.

### 2.4. Bioassay-Guided Fractionation of the Herba Cynomorii Ethanol Extract Utilizing an ATP-GC *In Situ* Assay as Activity Monitor

The ethanol extract of Herba Cynomorii was fractionated by silica gel column chromatography, with stepwise elution using a mixture of acetone and petroleum ether (3 : 7, 1 : 1 and 7 : 3; two bed volumes each), which was followed by absolute ethanol. Four crude fractions, termed, A1, A2, A3, and A4, were obtained. Based on the biological activity (refer to Section 3), A1 and A2 were grouped together, and the mixture was again subjected to silica gel column chromatography, with elution by a mixture of acetone and petroleum ether (3 : 7, v/v) to yield three subfractions: HCY1 (57 g), HCY2 (140 g), and HCY3 (16 g). All these fractions were concentrated under reduced pressure and stored at 4°C prior to use.

For the measurement of ATP-GC *in situ, *H9c2 cells were seeded at a density of 2.0 × 10^4^ cells/well in a 24-well culture plate, and cells in each well were allowed to grow to 60–80% confluence in a humidified incubator at 37°C within 2 days prior to drug treatment. Herbal extracts (dissolved in DMSO) were added to the medium to achieve the desired final concentrations (DMSO < 0.2%, v/v). After a 4 h incubation with the herbal extract or UA, the cells were subjected to the measurement of ATP-GC, as described by Leung and Ko [19].

### 2.5. HPLC-QQQ-MS/MS Analysis

HPLC analysis was performed using an Agilent RRLC 1200 series system (Agilent, Waldbronn, Germany), which was equipped with a degasser, a binary pump, an autosampler, and a thermostated column compartment. HCY2 (20 *μ*g/mL, dissolved in acetonitrile) or UA (5 *μ*g/mL, dissolved in acetonitrile) were separated on a Waters Atlantis C18 column (5 *μ*m id, 4.6 mm × 150 mm). The mobile phase was composed of acetonitrile (solvent A) and 0.1% formic acid in water (solvent B), using an isocratic gradient 78% (A) for 50 min. The flow rate was 0.4 mL/min. The column temperature was 25°C. The injection volume was 5 *μ*L. For the MS/MS analysis, an Agilent QQQ-MS/MS (6410A) equipped with an ESI ion source was operated in positive ion mode. The drying gas temperature was 325°C, with a gas flow of 10 L/min, nebulizer pressure at 35 psig, capillary voltage at 4000 V, and delta electro multiplier voltage at 400 V. Two suitable transition pairs were chosen for acquisition in MRM mode for UA. The fragmentor voltage and collision energy values were optimized to obtain the highest abundance. Agilent Mass Hunter workstation software (version B.01.00) was used for data acquisition and processing.

### 2.6. HPLC-UV Analysis

For quantitative analysis, HCY2 (1.4 mg/mL, dissolved in acetone and petroleum ether (3 : 7)) or UA was separated using an Agilent RRLC 1200 series system (Agilent, Waldbronn, Germany) consisting of an online degasser, two binary pumps, a high-performance SL autosampler, a thermostated column compartment, and a photodiode array UV-VIS detector. A mobile phase consisting of water (solvent A) and acetonitrile (solvent B) was applied for linear gradient elution (0 min 40% B, 50 min 100% B, 60 min 100% B) on an Agilent ZORBAX SB-Aq C18 column (5 *μ*m, 4.6 × 250 mm) at a flow rate of 1.0 mL/min and a temperature of 25°C. The detection wavelength was set at 205 nm. The quantitation of UA in the HCY2 fraction was determined from a calibration curve. A 5 mg/mL standard stock solution of UA was prepared in methanol. A serial dilution with methanol was made of each stock solution to prepare standard solutions at concentrations of 0.1, 0.2, 0.5, 1, and 2 mg/mL; 10 *μ*L of each standard solution was used for analysis and construction of a standard calibration curve.

### 2.7. Measurement of Substrate-Supported Mitochondrial Respiration Rates in Digitonin-Permeabilized Cells

Respiratory activity was measured polarographically with a Clark-type oxygen electrode (Hansatech Instruments, Norfolk, UK). H9c2 cells were seeded (2.0 × 10^6^ cells) in 100 mm culture plate. After stable attachment, cells were preincubated with herbal extracts or UA at desired final concentrations for 4 h at 37°C. After the incubation, the herbal extract- or UA-containing medium was aspirated and cells were collected by trypsinization followed by centrifugation. Cells were washed with phosphate buffered saline-A (PBS-A) twice, resuspended in assay buffer (120 mM KCl, 5 mM KH_2_PO_4_, 2 mM EGTA, 10 mM HEPES, 0.1 mM MgCl_2_, 0.5% BSA, pH 7.4), and stored at 37°C. An aliquot (1 mL) of suspended cells (1.5 × 10^6^ cells/mL) was placed in the air-tight liquid-phase oxygen electrode chamber. The system was maintained at 30°C using a constant temperature water-jacketing system. After equilibration, a nonionic detergent, digitonin (50 *μ*g/mL), was added and incubated for 3 min to permeabilize the cell membrane. This was followed by the addition of pyruvate (5 *μ*M), malate (2.5 *μ*M), and ADP (60 *μ*M) to allow mitochondrial state 3 respiration. Mitochondrial state 4 respiration was then induced by the addition of a specific complex V inhibitor, oligomycin (1 mg/mL). A typical polarographic recording of H9c2 cells respiration rates is shown in Figure 4(a).

### 2.8. Measurement of Cellular GSH Levels in H9c2 Cells

H9c2 cells were seeded (3.75 × 10^4^ cells/well) in 12-well culture plates. After stable attachment, cells were preincubated with herbal extracts or UA for increasing periods of time (2, 4, 8, 12, 16, and 24 h) at 37°C. Following incubation, reduced glutathione (GSH) levels were determined at each time point. Cellular GSH levels were determined enzymatically using DTNB (5, 5′-dithiobis-(2-nitrobenzoic acid) and glutathione reductase (GR), in a protocol modified from Griffith [20].

### 2.9. *In Vitro* Hypoxia/Reoxygenation-Induced Cell Apoptosis

Cells used for the experiment were seeded at 2.5 × 10^5^ cells on a 60 mm^2^ culture plate and allowed to grow overnight. After herbal extract or UA treatment, the cells were washed twice with Krebs-Ringer Bicarbonate buffer (KRB) containing 115 mM NaCl, 4.7 mM KCl, 2.5 mM CaCl_2_, 1.2 mM KH_2_PO_4_, 1.2 mM MgSO_4_, 24 mM NaHCO_3_, 10 mM HEPES, and (pH 7.4). Aliquots (2.5 mL) of KRB supplemented with 0.01% bovine serum albumin (BSA) were added to the cells immediately prior to the induction of hypoxia. A Billups-Rothenberg modular incubator chamber (Billups-Rothenberg, Inc., Del Mar, CA) was used to produce an *in vitro *hypoxia/reoxygenation (Hypo/Reoxy) challenge. In essence, cells were placed in the sealed chamber, and the chamber was flushed with nitrogen for 15 min at flow rate of 20 mL/min. After closing all sealable connectors, the chamber was transferred to an incubator and the cells in the chamber were subjected to a 2 h period of hypoxia at 37°C. Reoxygenation was initiated by opening the chamber and replacing the KRB with fresh DMEM medium. The cells were then cultured in the incubator under an atmosphere of 5% CO_2_ in air at 37°C for 16 h. The extent of apoptosis was quantified by measuring the activation of caspase 3 using a commercially available assay kit (Caspase 3 Assay Kit, Fluorimetric, SIGMA).

### 2.10. Animal Treatment

Adult male and female Sprague-Dawley rats (8–10 weeks; 250–300 g) were maintained under a 12 h dark/light cycle at about 22°C and allowed food and water *ad libitum*. Experimental protocols were approved by the Research Practice Committee at the Hong Kong University of Science & Technology. Animals were randomly divided into groups of 8 animals in each. In the treatment groups, male rats were intragastrically given HCY2 (dissolved/suspended in olive oil) at a daily dose of  16 or 48 mg/kg or UA (dissolved/suspended in olive oil) at a daily dose of 12 or 36 mg/kg for 14 consecutive days, respectively. Preliminary studies indicated that female rats responded to lower doses of HCY2 as assessed by the measurement of ATP-GC using isolated heart mitochondria. Female rats were given HCY2 (dissolved/suspended in olive oil) intragastrically at a daily dose of 2 or 5 mg/kg or UA (dissolved/suspended in olive oil) at a daily dose of 1.5 or 4 mg/kg for 14 consecutive days, respectively. Control animals received vehicle only. Twenty-four hours after the last dosing, animals were subjected to myocardial ischemia/reperfusion (I/R) challenge *ex vivo*.

### 2.11. Myocardial I/R Injury *Ex Vivo*

Hearts were quickly excised from phenobarbital-anesthetized rats and immediately immersed in ice-cold saline containing 50 unit/mL of heparin. The aorta was cannulated and the heart transferred to a warm, moist, perfusion chamber. The hearts were retrogradely perfused according to the Langendorff method [21]. After an initial 30 min of perfusion for equilibration, the isolated heart was subjected to a 40 min period of “no-flow” global ischemia followed by 20 min of reperfusion. Fractions of coronary effluent, collected at 1 min intervals, were obtained both during the course of equilibration and during the subsequent reperfusion. The coronary fractions were immediately placed on ice and assayed for lactate dehydrogenase (LDH) activity. The extent of LDH leakage during the reperfusion period, an indirect index of myocardial injury, was estimated by computing the area under the curve (AUC) of a graph plotting the percentage LDH activity (with respect to the mean preischemic value measured during the equilibration period) against the reperfusion time (1–20 min), as described in [21]. The extent of damage was expressed in arbitrary units. Non-I/R hearts were perfused for 90 min. After either non-I/R or I/R procedures, heart ventricular tissue samples were obtained and subjected to biochemical analysis.

### 2.12. Measurement of Tissue ATP Level

Samples of perfused and I/R ventricular tissue (approximately 70 mg) were homogenized with 5% perchloric acid (PCA) (4 *μ*L/mg tissue) at 4°C. Following centrifugation at 2150 ×g for 10 min at 4°C, the supernatant was diluted 5-fold with 5% PCA. An aliquot (120 *μ*L) of the supernatant was neutralized with 90 *μ*L of 1.4 M KHCO_3_, followed by mixing and centrifugation. The resulting supernatant was analyzed for ATP content using a bioluminescence assay (ATPlite, Perkin Elmer, Inc. USA).

### 2.13. Preparation of Mitochondrial Fractions

Mitochondrial fractions were prepared by differential centrifugation, as previously described in [21]. In brief, myocardial ventricular tissue was homogenized in 10 volumes of ice-cold mannitol/sucrose buffer (210 mM mannitol, 70 mM sucrose, 5 mM HEPES, 1 mM EGTA, pH 7.4) using a Teflon-glass homogenizer. The homogenate was centrifuged at 600 ×g for 10 min, and the pellet containing nuclear and cell debris was discarded. The supernatant was centrifuged at 9200 ×g for 30 min to obtain the mitochondrial fraction. The mitochondrial pellet was suspended in ice-cold mannitol/sucrose buffer.

### 2.14. Biochemical Analyses in Rat Heart Mitochondria

The mitochondrial ATP-GC of rat hearts was measured *ex vivo* by a method previously described in [14]. Mitochondrial GSH level and the GSH/oxidized glutathione (GSSG) ratio were measured using an enzymatic method of Griffith [20]. Mitochondrial GR activity was measured by monitoring the oxidation of NADPH spectrophotometrically, as previously described in [22].

### 2.15. Protein Assay

Protein concentrations of mitochondrial fractions and cell lysates were determined using a Bio-Rad protein assay kit using bovine serum albumin as standard.

### 2.16. Statistical Analysis

All data were expressed as mean ± standard error of the mean (SEM). They were analyzed by one-way analysis of variance (ANOVA). Post hoc multiple comparisons were done with LSD. A difference was considered statistically significant if *P *value < 0.05.

## 3. Results

### 3.1. Bioassay-Guided Fractionation of Herba Cynomorii

**Figure 1:**
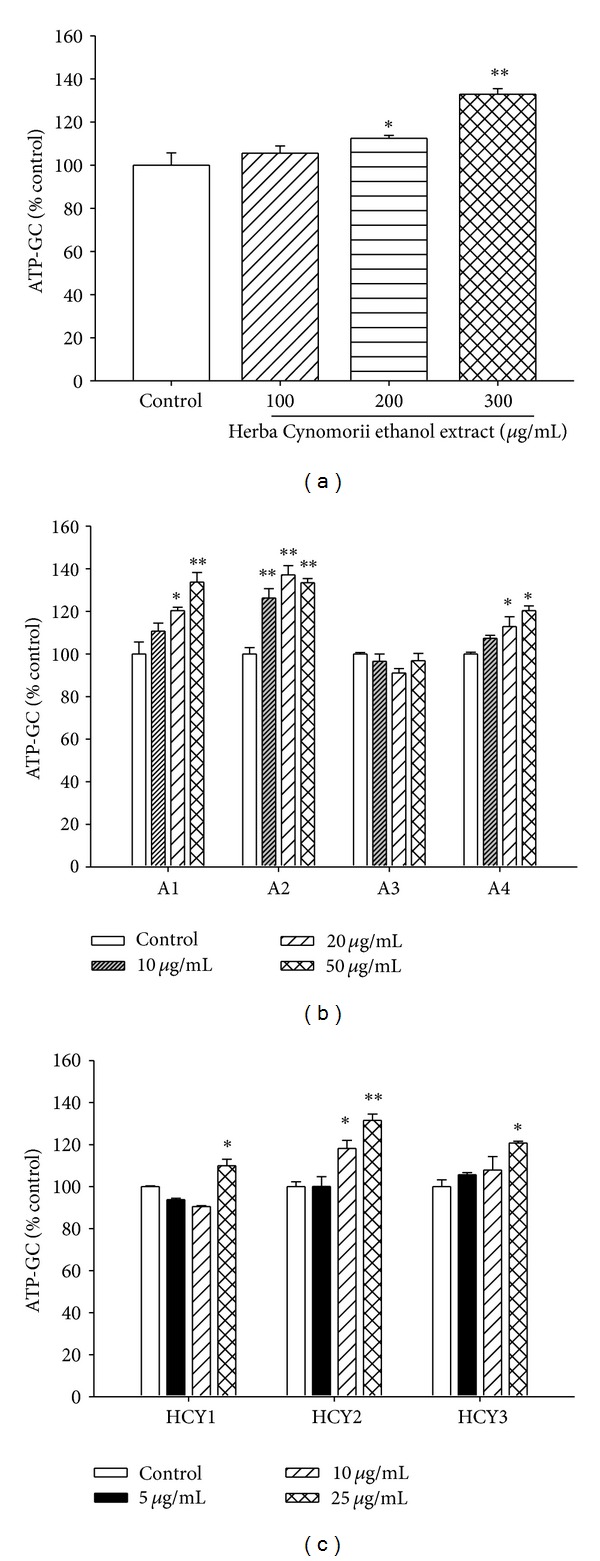
Effect of ethanol extract and different fractions of Herba Cynomorii on ATP-GC in H9c2 cells. Cells were incubated with herbal extracts at the indicated concentrations for 4 h. ATP-GC was measured as described in Section 2. Data were expressed as the percentage of nonpreincubated control values [AUC_2_ = 949 ± 22 (SEM)]. Values given are means ± SEM, with *n* = 6. *P < 0.05 and **P < 0.01, when compared with the control group without herbal extract preincubation.

The fractionation scheme of the Herba Cynomorii ethanol extract (1.82 kg) is depicted in supplementary materials (see Supplementary Materials available online at http://dx.doi.org/10.1155/2013/924128). As shown in Figure 1(a), preincubation with the Herba Cynomorii ethanol extract increased ATP-GC in H9c2 cells in a concentration-dependent manner, with the maximum extent of stimulation being 33% at the concentration of 300 *μ*g/mL. Silica gel column chromatography of the Herba Cynomorii ethanol extract yielded four fractions (A1–A4), which were subjected to ATP-GC assay.  Figure 1(b) shows that preincubating the cells with fractions A1 or A2 significantly enhanced ATP-GC in H9c2 cells, with the maximum extent of stimulation being 37% at 20 *µ*g/mL and 34% at 50 *μ*g/mL, respectively. Preincubation with fraction A4 also significantly increased ATP-GC in H9c2 cells, but to a lesser extent than that of fractions A1 or A2, the extent of stimulation being 20%. In contrast, preincubating H9c2 cells with fraction A3 produced no detectable effect on ATP-GC, when compared with the control group. Silica gel thin layer chromatography (TLC) analysis of fractions A1 and A2 revealed overlapping spots (data not shown). Fractions A1 and A2 were combined and subjected to refractionation by silica gel column chromatography. Three semipurified fractions (HCY1, HCY2 and HCY2) were obtained at yields of 3.1, 7.7, and 0.9%, respectively. As shown in Figure 1(c), preincubation with fraction HCY2 increased ATP-GC (5–25 *μ*g/mL) in H9c2 cells in a concentration-dependent manner, with the maximum stimulation being 32% at 25 *μ*g/mL. Preincubation with fractions HCY1 and HCY3 also maximally stimulated ATP-GC at 25 *μ*g/mL, with the degrees of enhancement being 10% and 21%, respectively. Thus, HCY2, which was the most abundant of the three semipurified fractions, was the most effective in stimulating ATP-GC in H9c2 cells. 

### 3.2. Identification and Quantitation of UA in HCY2 and the Effect of UA on ATP-GC

**Figure 2:**
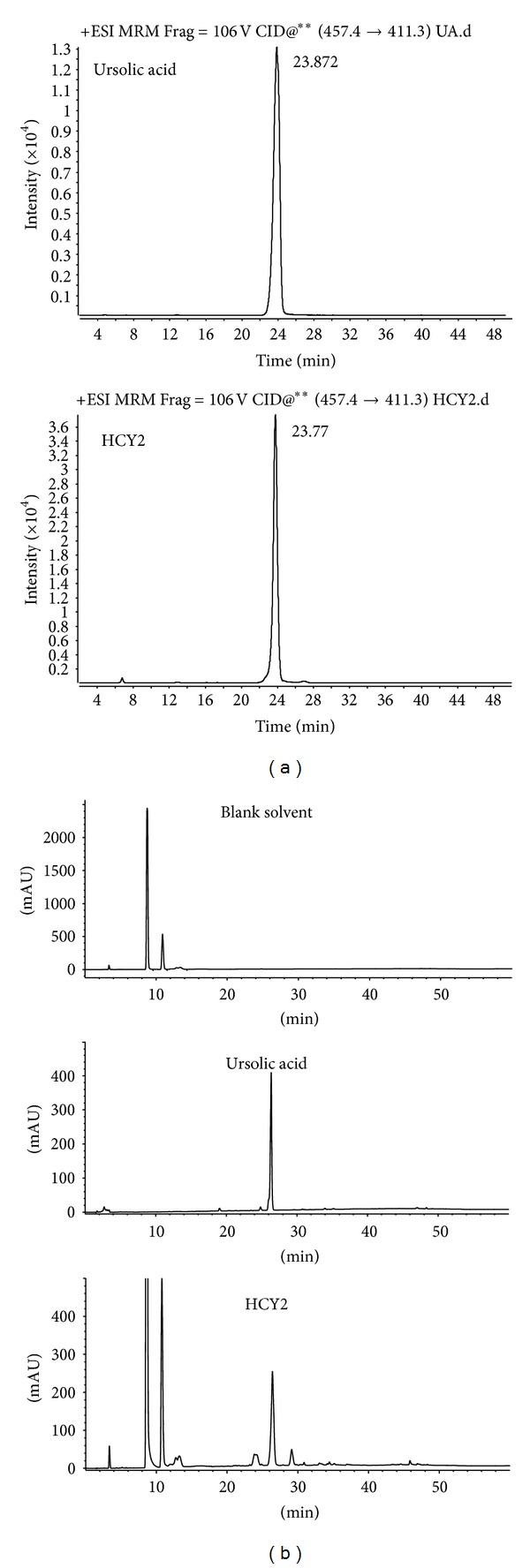
Typical HPLC-QQQ MS/MS chromatogram and HPLC-UV chromatogram of UA and HCY2. The chromatographic method is described in Section 2. (a) Selected transitions from precursor ions to product ions used for qualitative analysis of UA in HCY2 after optimization by MRM. (b) The quantitative analysis of UA was made by HPLC-UV (205 nm).

**Figure 3:**
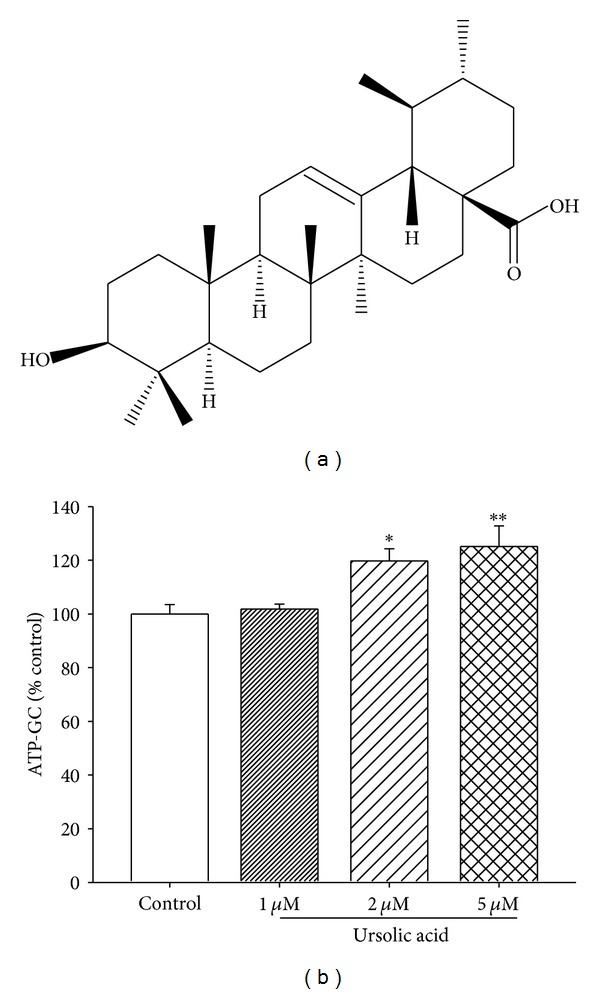
(a) Chemical structure of UA. (b) Effect of UA on ATP-GC in H9c2 cells. Cells were incubated with UA at the indicated concentrations for 4 h. ATP-GC was measured as described in Section 2. Data are expressed as the percentage of nonpreincubated control values [AUC_2_ = 750 ± 26 (SEM)]. Values given are means ± SEM, with *n* = 6. *P < 0.05 and **P < 0.01, when compared with the control group without herbal extract preincubation.

By using LC-MS/MS analysis which involved the comparison of fragmentation and retention times with those of standards in the multiple-reaction monitoring (MRM) scan mode (Figure 2(a)), the major component in HCY2 was identified as UA (see chemical structure in Figure 3(a)). The retention time of UA was 23.7 min. The MS/MS transition of UA (MW 456) monitored in the positive ion mode was *m/z* 457 [M + 1] → *m/z* 411 [M–COOH].

As shown in Figure 2(b), a baseline separation of UA was achieved by reverse phase-HPLC with UV detection. The calibration curve of UA was linear and the regression equation of the peak area (*y*) as a function of concentration (*x*) was *y* = 6609.4*x* − 271.4  (*r* = 0.999). The content of UA in HCY2 was estimated to be 75% (w/w).

Preincubation of H9c2 cells with UA increased the ATP-GC in a concentration-dependent manner, with the maximum stimulation being 25% (5 *μ*M), when compared with the control (Figure 3(b)).

### 3.3. Effect of HCY2 or UA on Mitochondrial Respiration in H9c2 Cells

**Figure 4:**
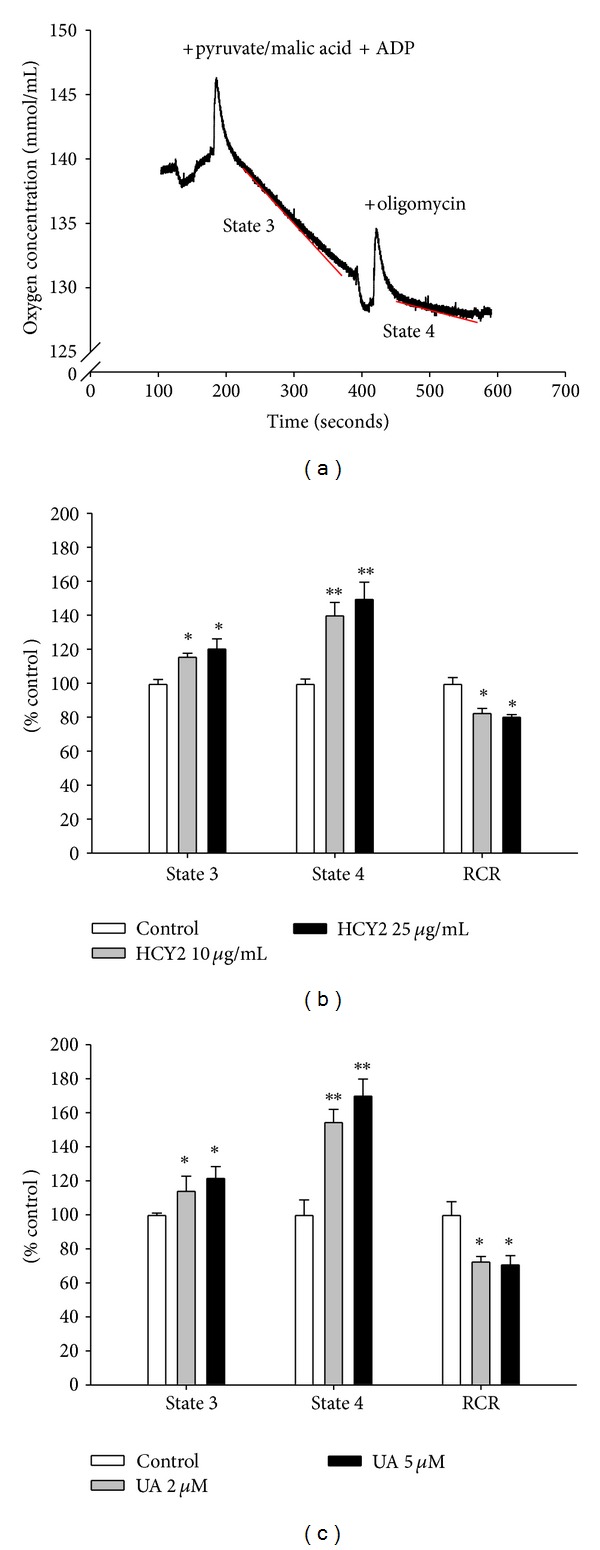
Effect of HCY2 and UA on mitochondrial respiration in H9c2 cells. Cells were preincubated with HCY2 and UA at the indicated concentrations for 4 h and subjected to the measurement of oxygen consumption. Typical oxygen-electrode traces of cellular respiration in H9c2 cells are shown in (a). The effects of HCY2 (b) or UA (c) preincubation on mitochondrial respiration were examined. Data are expressed in percent control with respect to the nonpreincubated control (state 3 respiration rate = 3.34 ± 0.06 nmol O_2_/min, state 4 respiration rate = 0.78 ± 0.06 nmol O_2_/min, RCR = 4.37 ± 0.28). Values given are means ± SEM, with *n* = 3. *P < 0.05 and **P < 0.01, when compared with the control group without drug or herbal extract preincubation.

Figure 4(b) shows that preincubation with HCY2 or UA increased state 3 respiration in H9c2 cells in a concentration-dependent manner, with the extent of stimulation induced by HCY2 (25 *µ*g/mL) being similar to that of UA (5 *μ*M) (~21%). State 4 respiration was also increased by 50 and 70%, respectively, in HCY2 and UA preincubated cells (Figure 4(c)). The ratio of state 3/state 4 respiration rate, which is also referred to as the respiration control ratio (RCR) that reflects the coupling efficiency of mitochondria during respiration, was decreased by 19% (HCY2) and 29% (UA) at the highest concentration tested. 

### 3.4. Effect of HCY2 or UA on Cellular GSH Levels in H9c2 Cells

**Figure 5:**
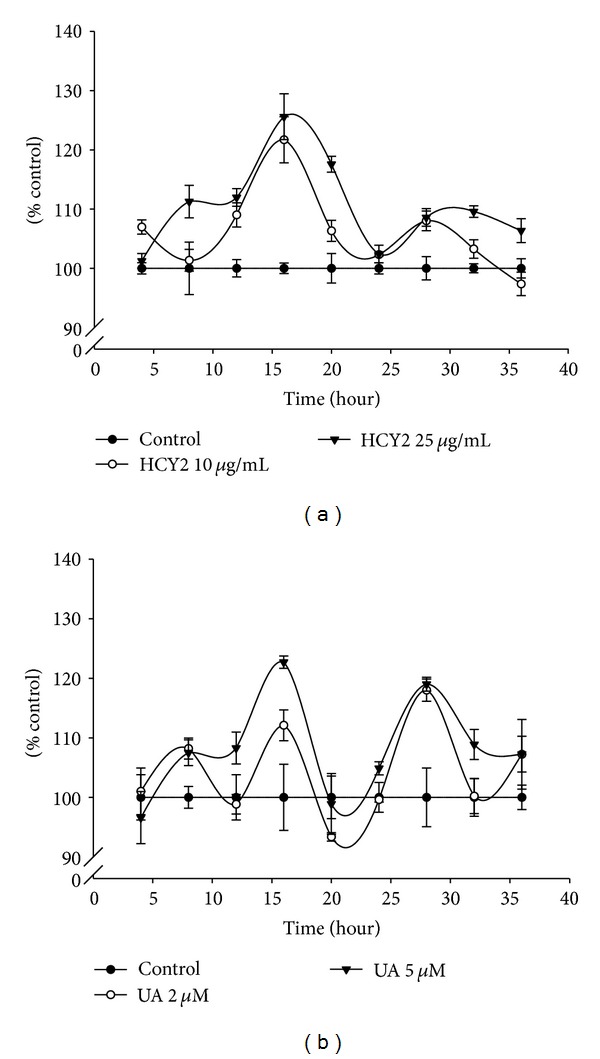
Time course of HCY2/UA-induced changes in cellular GSH level in H9c2 cells. HCY2 or UA was added at the indicated concentrations. Reduced glutathione (GSH) levels were measured as described in Section 2. Data are expressed as the percentage of non drug incubated parallel control values (initial control GSH level (a) = 30.6 ± 2.87 nmol/mg protein, (b) = 27.1 ± 1.90 nmol/mg protein) at various periods of herbal extract or drug incubation. Values given are means ± SEM, with *n* = 4.

As shown in Figure 5, exposure to HCY2 (10 or 25 *µ*g/mL) or UA (2 or 5 *μ*M) produced time-driven cyclic variations in cellular GSH levels in H9c2 cells, with concentration-dependent increases in the amplitude of oscillation (21–26% and 12–23%, resp.), when compared with the control. 

### 3.5. Effect of HCY2 or UA on Hypo/Reoxy-Induced Apoptosis in H9c2 Cells

**Figure 6:**
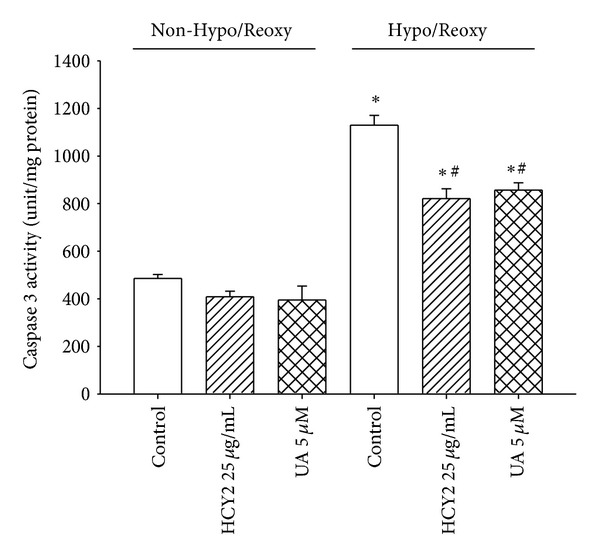
Effect of HCY2/UA preincubation on hypoxia/reoxygenation-induced apoptosis in H9c2 cells. Cells were preincubated with HCY2 (25 *μ*g/mL) or UA (5 *μ*M) for 4 h and then subjected to hypoxia/reoxygenation (Hypo/Reoxy) challenge, as described in Section 2. The extent of apoptotic cell death was assessed by the measurement of caspase-3 activation. Values given are means ± SEM, with *n* = 3. “∗” significantly different from the unchallenged control; “#” significantly different from Hypo/Reoxy-challenged control.

Hypoxia/reoxygenation caused apoptosis in H9c2 cells, as evidenced by a significant increase (1.2 fold) of caspase-3 activity in Hypo/Reoxy-challenged cells, when compared with unchallenged controls. Prior exposure to HCY2 (25 *μ*g/mL) or UA (5 *μ*M) for 4 h significantly suppressed the Hypo/Reoxy-induced increase in caspase-3 activity, with the degrees of protection being approximately 36 and 28%, respectively (Figure 6). 

### 3.6. Effect of HCY2 or UA on Myocardial I/R Injury in Rats **Ex Vivo**

**Figure 7:**
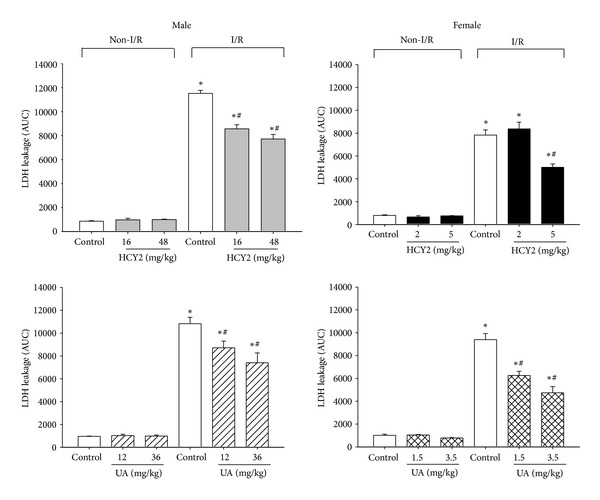
Effect of HCY2/UA pretreatment on myocardial I/R injury in male and female rats *ex vivo*. Animals were orally treated with vehicle (olive oil) or HCY2/UA (at the indicated doses) for 14 consecutive days. Isolated hearts were subjected to I/R challenge as described in Section 2. The extent of tissue injury was assessed by the measurement of LDH leakage. Each bar represents mean ± SEM, with *n* ≥ 3. “∗” significantly different from the non-I/R control; “#” significantly different from I/R-challenged control.

I/R caused tissue damage in isolated-perfused rat hearts, as evidenced by the significant increase in LDH leakage (9- to 11-fold). Hearts from male rats were more susceptible to I/R injury than those from females, as indicated by a greater extent of LDH leakage (11-fold versus 9-fold) (Figure 7). HCY2 pretreatment (16 or 48 mg/kg) dose dependently protected against myocardial I/R injury in male rats, with the degrees of protection being 27 and 37%, respectively. UA pretreatment (12 or 36 mg/kg) also protected against myocardial I/R injury in male rats, with the degrees of protection being 22 and 35%, respectively. As for female rats, although HCY2 pretreatment at a dose of 2 mg/kg did not produce any detectable effect, there was significant protection against I/R injury at a dose of 5 mg/kg, with the degree of protection being 39%. Pretreatment with UA (1.5 or 3.5 mg/kg) resulted in protection against myocardial I/R injury in female rats, with the degree of protection being 38 and 53%, respectively. 

### 3.7. Effect of HCY2 or UA on Mitochondrial ATP-GC in Rat Hearts

**Figure 8:**
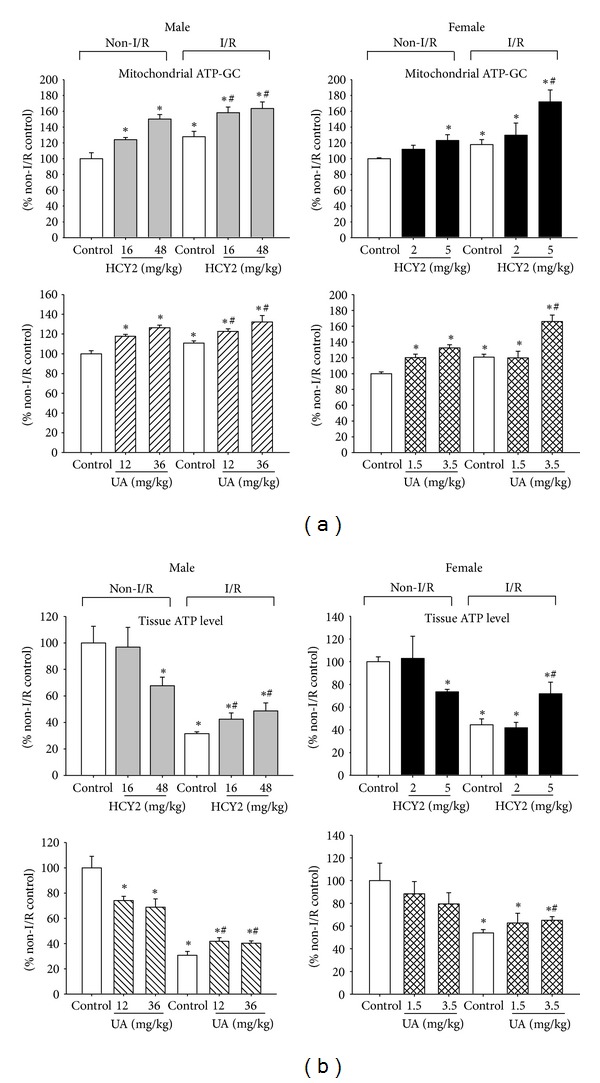
Effect of HCY2/UA pretreatment on (a) mitochondrial ATP-GC and (b) tissue ATP levels in control perfused and ischemic-reperfused hearts. Biochemical assays of ATP-GC and tissue ATP level were performed as described in Section 2. Data are expressed as the percentage of non-I/R control values. The values of ATP-GC of non I/R control were [AUC_2_ = 1000 ± 24  (male HCY2), 1000 ± 36 (male UA), 1000 ± 11 (female HCY2), and 1000 ± 21 (female UA)]. The values of tissue ATP level of non-I/R control were 1365 ± 183 (male HCY2), 1024 ± 76 (male UA), 1641 ± 70 (female HCY2), and 1558 ± 240 (female UA) nmol/mg protein. Each bar represents mean ± SEM, with *n* ≥ 3. “∗” significantly different from the non-I/R control; “#” significantly different from I/R-challenged control.

As shown in Figure 8(a), under non-I/R conditions, HCY2 pretreatment (16 or 48 mg/kg) significantly increased mitochondrial ATP-GC, with the extent of stimulation being 24–50% in male rat hearts. HCY2 pretreatment at a dose of 5 mg/kg significantly increased mitochondrial ATP-GC by 23% in female rat hearts. UA pretreatment (12 or 36 mg/kg for males; 1.5 or 3.5 mg/kg for females) dose dependently enhanced mitochondrial ATP-GC in both male and female rat hearts under non-I/R conditions, with the extent of stimulation being 17–32%.

An increase in ATP-GC was also observed after I/R challenge in both male and female rat hearts, with the extent being 10 to 20% when compared with the non-I/R controls. Under I/R conditions, HCY2 pretreatment (16 or 48 mg/kg) further increased mitochondrial ATP-GC (21–36%) in male rats, when compared with challenged controls. HCY2 pretreatment at a dose of 5 mg/kg enhanced mitochondrial ATP-GC by 54% in female rat hearts subjected to I/R challenge, when compared with unpretreated and challenged hearts. In male rats, UA pretreatment (12 or 36 mg/kg) dose dependently increased mitochondrial ATP-GC by 13 and 22%, respectively, under I/R conditions, when compared with I/R-challenged controls. UA pretreatment at a dose of 3.5 mg/kg also increased mitochondrial ATP-GC by 46% in female rat hearts under I/R conditions. 

### 3.8. Effect of HCY2 and UA on Tissue ATP Level in Rat Hearts

Under non-I/R condition, HCY2 pretreatment at 48 mg/kg significantly decreased tissue ATP level by 32% when compared with the control group in male rats. In female rats, HCY2 pretreatment at a dose of 5 mg/kg significantly decreased tissue ATP level by 26%. UA (12, 36 mg/kg) dose dependently reduced tissue ATP level in male rat hearts, with the extent of reduction being 26–31%. However, UA (1.5, 3.5 mg/kg) only caused a slight decrease in tissue ATP level in female rat hearts (11 and 20%) under non-I/R condition. The I/R challenge also resulted in a dramatic decrease in tissue ATP level, with the extent of decrease being 46–70% when compared with the non-I/R control. Both HCY2 and UA partially attenuated the decrease in tissue ATP level by 10–27% (Figure 8(b)). 

### 3.9. Effect of Pretreatment with HCY2 or UA on Mitochondrial Glutathione Redox Status in Rat Hearts

**Figure 9:**
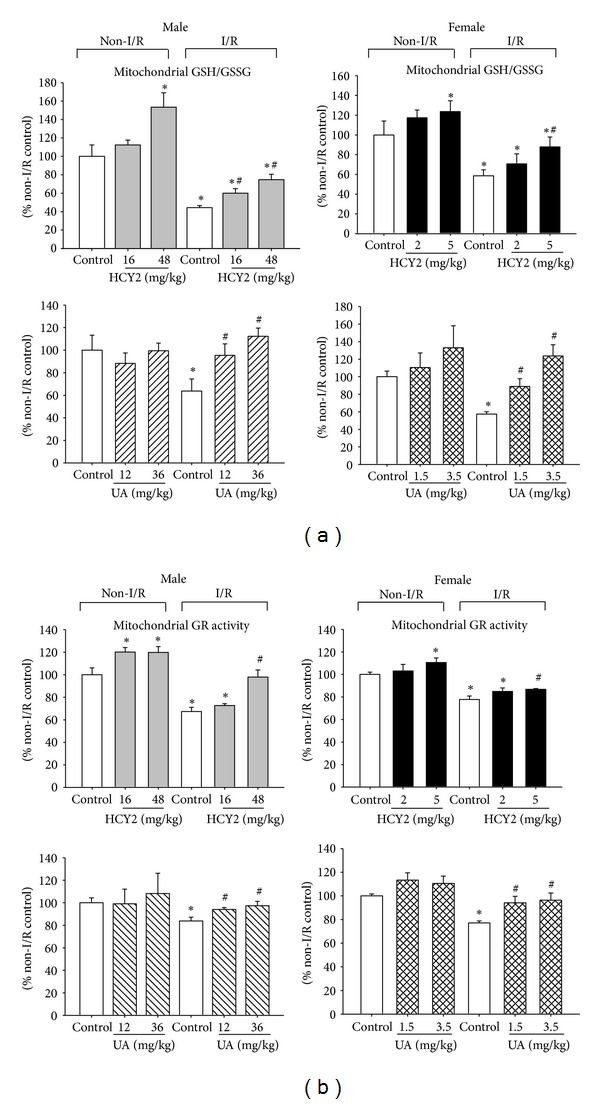
Effect of HCY2/UA pretreatment on (a) mitochondrial GSH/GSSG and (b) GR activity in control perfused and ischemic-reperfused hearts. The ratio of mitochondrial GSH/GSSG and the activity of GR were measured as described in Section 2. The values of mitochondrial GSH/GSSG of non-I/R control were 5.0 ± 0.21 (male HCY2), 4.4 ± 0.58 (male UA), 7.0 ± 0.99 (female HCY2), and 6.0 ± 0.38 (female UA). The values of mitochondrial GR activity of non-I/R control were 2.64 ± 0.11 (male HCY2), 2.38 ± 0.14 (male UA), 2.91 ± 0.06 (female HCY2), and 1.71 ± 0.03 (female UA) mU/mg protein. Each bar represents mean ± SEM, with *n* ≥ 3. “∗” significantly different from the non-I/R control; “#” significantly different from I/R-challenged control.

Under non-I/R conditions, HCY2 pretreatment at 48 mg/kg significantly increased the mitochondrial GSH/GSSG ratio in male rats, with the extent of enhancement being 54%. In female rats, HCY2 pretreatment at a dose of 5 mg/kg significantly increased the mitochondrial GSH/GSSG ratio (by 24%), when compared with non-challenged controls. However, UA pretreatment did not produce any detectable changes in the mitochondrial GSH/GSSG ratio in either male or female rat hearts in the absence of I/R challenge. I/R caused a significant decrease in the mitochondrial GSH/GSSG ratio (by 40–60%) in both male and female rat hearts. HCY2 pretreatment significantly reduced the change in the GSH/GSSG ratio following I/R challenge by 16% (16 mg/kg) and 30% (48 mg/kg) in male rat hearts as well as 30% (5 mg/kg) in female rat hearts. UA also significantly reduced the I/R-associated change in the GSH/GSSG ratio by 32% (12 mg/kg) and 50% (36 mg/kg) in male rat hearts as well as 30% (1.5 mg/kg) and 66% (3.5 mg/kg) in female rats (Figure 9(a)). 

The enhancement of glutathione redox status was accompanied by increases in mitochondrial GR activities in hearts of HCY2- and UA-pretreated animals. In male rats, HCY2 pretreatment increased the mitochondrial GR activity by 20% in the absence of I/R. In female rats, HCY2 pretreatment at a dose of 5 mg/kg caused a significant increase in mitochondrial GR activity by 11%. UA pretreatment did not produce any detectable changes in mitochondrial GR activity in either male or female rat hearts in the absence of I/R challenge. I/R caused a significant decrease in mitochondrial GR activity by 17–33%, when compared with the non-I/R controls. HCY2 pretreatment at a dose of 48 mg/kg for male rats and 5 mg/kg for female rats attenuated the I/R-induced decrease in mitochondrial GR activity by 30 and 10%, respectively. UA pretreatment also reduced the decrease in mitochondrial GR activity by 10–20% in both male and female rat hearts (Figure 9(b)).

## 4. Discussion

The decline in mitochondrial functional capacity can lead to a deficit in cellular energy supply, especially in organs such as the heart that has a large energy demand. An accumulated body of experimental and clinical evidence has shown that an impairment in energy metabolism resulting from mitochondrial dysfunction plays a major role in the pathogenesis of various heart diseases [23]. An impairment in myocardial oxidative phosphorylation was observed in an experimental model of heart failure, which revealed a dramatic decrease in the maximal ATP generating capacity of mitochondria [24]. Therefore, the maintenance of mitochondrial functional integrity is likely to play an important role in the preventive health of the heart.

Herba Cynomorii is one of the most commonly used “Yang-tonic” herbs for treating what is referred to as “kidney deficiency” in Chinese medicine. Previous studies have shown that administration of Herba Cynomorii enhances mitochondrial ATP-GC both *in vitro *in H9c2 cells and *ex vivo *in rat hearts, presumably through enhancing mitochondrial electron transport [13, 18]. Using an ATP-GC assay as a monitor of biological activity in the fractionation of a Herba Cynomorii ethanol extract, HCY2 was found to be the most abundant fraction with ATP-GC-stimulating activity in H9c2 cells. While Herba Cynomorii was found to contain triterpenes, steroids, flavonoids, phenolic acid, lignans, and polysaccharides [25–28], silica gel TLC analysis of HCY2 showed a major violet spot revealed after spraying with sulfuric acid in ethanol, indicative of steroid- or triterpene-like compounds (data not shown). Further chemical analysis using LC-MS/MS indicated that HCY2 contained UA as the major constituent, which amounted to 75% (w/w), as determined by HPLC analysis using authentic UA as standard. This finding is corroborated by the observation that UA (1–5 *μ*M) stimulated ATP-GC in H9c2 cells in a concentration-dependent manner, albeit to a lesser degree when compared with that of HCY2 at doses of 10–25 *μ*g/mL. However, UA at 17.5 and 45 *μ*M (concentrations equivalent to 10 and 25 *μ*g/mL HCY2, resp.) produced cytotoxicity in H9c2 cells (data not shown).

The measurement of ATP-GC using malate and glutamate as substrates is an indirect measure of state 3 mitochondrial respiration [29]. Consistently, preincubation with both HCY2 or UA significantly increased state 3 respiration rate in H9c2 cells, as assessed by oxygen consumption. HCY2 and UA preincubations also increased state 4 respiration rate in H9c2 cells, with a resultant reduction in the respiration control ratio, indicative of mitochondrial uncoupling. Mitochondrial uncoupling can be induced by chemical uncouplers as well as by activating uncoupling proteins (UCPs) [30], with resultant increases in oxygen consumption secondary to an increase in electron transport [31]. UA, the major component of HCY2, was found to induce mitochondrial uncoupling in isolated rat heart mitochondria [32]. Such an uncoupling effect produced by the UA-enriched HCY2 could stimulate electron transport in mitochondria, thereby enhancing ATP-GC, which was measured under optimal experimental conditions. In this regard, an uncoupler such as dinitrophenol was found to not only induce uncoupling, but also to stimulate ADP-stimulated respiration rate (i.e., state 3) in liver mitochondria [33]. In addition, the overexpression of UCP4 was shown to facilitate ATP production using succinate as substrate, which was also associated with an increased respiration rate, in neuroblastoma cells [34, 35].

GSH is regarded as the first line of defense in preventing cellular oxidative damage. Under the present experimental conditions, incubations with HCY2 or UA were both found to induce a time-dependent cyclic variation of cellular GSH levels in H9c2 cells, indicative of upregulation of glutathione redox cycling. As also observed in Herba Cistanches-treated H9c2 cells [36], the activation of glutathione redox cycling is presumably related to the interplay between HCY2/UA-induced reactive oxygen species (ROS) generation, an event secondary to the increased electron transport, and the GR-catalyzed and NADPH-mediated regeneration of GSH. Given that increased formation of ROS within mitochondria can cause an adaptive response mediated by the activation of nuclear factor-erythroid 2 p45-related factor 2 [37, 38], HCY2/UA may therefore positively regulate GR, which in turn catalyzes the regeneration of GSH from GSSG, leading to the cyclic change in cellular GSH levels.

The enhancement of HCY2/UA-induced glutathione redox cycling was associated with protection against hypoxia/reoxygenation-induced apoptosis in H9c2 cells. Oxidative stress arising from an excessive production of ROS caused by Hypo/Reoxy challenge can cause cellular injury, which mimics the pathophysiological condition of I/R injury [39, 40]. It has been reported that the ethyl acetate fraction of Herba Cynomorii significantly attenuates staurosporine-induced apoptosis in SK-N-SH neuroblastoma cells [41]. UA was also found to reduce both intracellular ROS levels and the rate of H_2_O_2_-induced apoptosis in leukemic cells [42]. To confirm the protective effect of HCY2/UA on oxidant injury, its effects were examined in a rat model of myocardial I/R injury. In the absence of I/R, both HCY2 and UA pretreatment invariably enhanced mitochondrial functional capacity in hearts of both male and female rats. On the other hand, HCY2/UA pre-treatment reduced tissue ATP levels in both male and female rat hearts, which indicated the involvement of uncoupled respiration. The observation of an increase in mitochondrial ATP-GC and a decrease in tissue ATP content would seem to be contradictory. While the mitochondrial ATP-GC reflects the ability of isolated mitochondria to generate ATP *in situ *under optimal assay conditions, the tissue ATP level indicates the steady state energy status of rat hearts under normal conditions. The enhancement of mitochondrial functional capacity was associated with an increase in mitochondrial glutathione antioxidant status, as was the case in H9c2 cells. Reperfusion of the previously ischemic myocardium triggers a burst of ROS formation, resulting in oxidative tissue damage [43]. The beneficial effects of HCY2 and UA pretreatment became more evident under conditions of I/R, where significant protection against I/R-induced tissue damage was observed. The cardioprotection afforded by HCY2 and UA pretreatments was associated with an enhancement of mitochondrial functional capacity. I/R *per se* also stimulated ATP-GC, which could reflect an adaptive response of rat heart mitochondria during reperfusion for maintaining a sufficient supply of energy for metabolism. The decreased tissue ATP content after I/R challenge was probably due at least partly to impaired oxidative phosphorylation and electron transport during postischemic reperfusion. HCY2 or UA pretreatment both ameliorated the extent of ATP depletion, presumably at least in part due to an enhancement of ATP-GC. The increase in mitochondrial functional capacity in hearts of HCY2/UA-pretreated rats was paralleled by a significant attenuation of the I/R-induced decrease in mitochondrial GSH/GSSG ratio as well as in mitochondrial GR activity, indicative of enhancement of glutathione redox cycling.

Interestingly, HCY2/UA pretreatment produced more prominent protection against myocardial I/R injury in female than in male rat hearts. While the differential susceptibility of males and females to tissue oxidative damage, as seen in experimental and clinical observations, is likely attributed to biochemical event(s) secondary to the action of estrogens [44, 45], the gender-dependent myocardial protection afforded by HCY2 or UA pretreatment may be related to an interaction between sex hormones and HCY2/UA on mitochondrial energetics. In this regard, male sex hormones (dihydrotestosterone and testosterone) have been shown to reverse protonophore-induced weak uncoupling (i.e., recoupling) in rat liver, heart, and skeletal mitochondria, thereby increasing the mitochondrial membrane potential and lowering respiration rate [46]. In contrast, estrogen showed no such recoupling effect [46]. Given the proposed involvement of mitochondrial uncoupling in the cardioprotective effects afforded by HCY2 and UA, it is possible that male sex hormones could largely compromise the uncoupling induced by HCY2 and UA in male rats, resulting in a decrease in the extent of enhancement in ATP-GC and antioxidant response. 

In conclusion, HCY2 and its major constituent, UA, increased mitochondrial functional capacity and upregulated cellular/mitochondrial antioxidant status, thereby protecting against oxidant-induced cellular/tissue injury in both *in vitro* and *ex vivo *assays. This beneficial effect produced by Herba Cynomorii is likely mediated by mitochondrial uncoupling. Even though several disparities between the effects of HCY2 and UA on mitochondrial glutathione antioxidant status were found, UA seems to be a “Yang-invigorating” ingredient of Herba Cynomorii, particularly in the HCY2 extract. Further investigation on the beneficial effects of HCY2 extract on other organs, such as the liver, kidney, and brain, would seem to be warranted.

## Supplementary Material

The ethanol extract of Herba Cynomorii (1.82 kg) was fractionated by silica gel column chromatography, with stepwise elution using a mixture of acetone and petroleum ether (3:7, 1:1 and 7:3; two bed-volumes each), which was followed by absolute ethanol. Four crude fractions, termed, A1, A2, A3 and A4, were obtained. Based on the biological activity (refer to Section 3), A1 and A2 were grouped together and the mixture was again subjected to silica gel column chromatography, with elution by a mixture of acetone and petroleum ether (3:7, v/v) to yield three sub-fractions: HCY1 (57 g), HCY2 (140 g) and HCY3 (16 g).
