# Supplementary material for: Ursolic-Acid-Enriched Herba Cynomorii Extract Protects against Oxidant Injury in H9c2 Cells and Rat Myocardium by Increasing Mitochondrial ATP Generation Capacity and Enhancing Cellular Glutathione Redox Cycling, Possibly through Mitochondrial Uncoupling
Source: Evid Based Complement Alternat Med. 2013 Apr 10;2013:924128. doi: 10.1155/2013/924128 (PMC3638637; doi:10.1155/2013/924128)
Supplement: Supplementary file 1 — The ethanol extract of Herba Cynomorii (1.82 kg) was fractionated by silica gel column chromatography, with stepwise elution using a mixture of acetone and petroleum ether (3:7, 1:1 and 7:3; two bed-volumes each), which was followed by absolute ethanol. Four crude fractions, termed, A1, A2, A3 and A4, were obtained. Based on the biological activity (refer to Section 3), A1 and A2 were grouped together and the mixture was again subjected to silica gel column chromatography, with elution by a mixture of acetone and petroleum ether (3:7, v/v) to yield three sub-fractions: HCY1 (57 g), HCY2 (140 g) and HCY3 (16 g). [file 924128.f1.pdf]

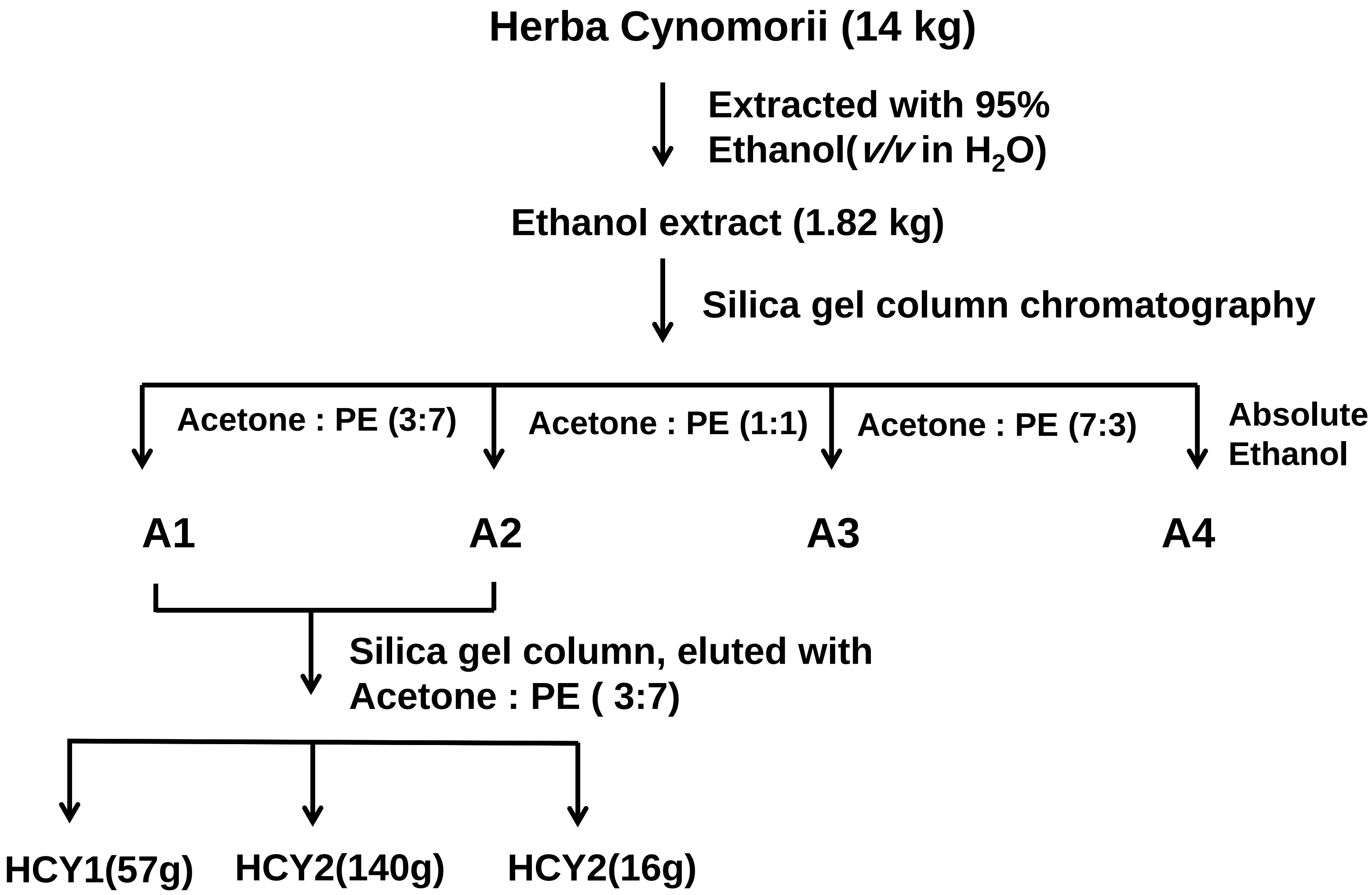
Herba Cynomorii (14 kg)
Extracted with 95% Ethanol(v/v in H_2O)
Ethanol extract (1.82 kg)
Silica gel column chromatography
Acetone : PE (3:7)
Acetone : PE (1:1)
Acetone : PE (7:3)
Absolute Ethanol
A1
A2
A3
A4
Silica gel column, eluted with Acetone : PE (3:7)
HCY1(57g)
HCY2(140g)
HCY2(16g)
